# 3-Nitro-1*H*-1,2,4-triazole

**DOI:** 10.1107/S1600536810049287

**Published:** 2010-12-04

**Authors:** Madhukar Hemamalini, Hoong-Kun Fun

**Affiliations:** a X-ray Crystallography Unit, School of Physics, Universiti Sains Malaysia, 11800 USM, Penang, Malaysia

## Abstract

The asymmetric unit of the title compound, C_2_H_2_N_4_O_2_, contains two crystallographically independent mol­ecules in which the triazole rings are essentially planar, with maximum deviations of 0.003 (1) Å in both molecules. The dihedral angle between the two 1*H*-1,2,4-triazole rings is 56.58 (5)°. In the crystal, mol­ecules are linked *via* inter­molecular N—H⋯N and C—H⋯O hydrogen bonds, forming a supra­molecular chain along the *b* axis.

## Related literature

For details and applications of 1*H*-1,2,4-triazole derivatives, see: Desenko (1995 ▶); Vos *et al.* (1983 ▶); van Albada *et al.* (1984 ▶); Al-Kharafi *et al.* (1986 ▶); Gupta & Bhargava (1978 ▶); Jones *et al.* (1965 ▶); Bennur *et al.* (1976 ▶). For the stability of the temperature controller used in the data collection, see: Cosier & Glazer (1986 ▶).
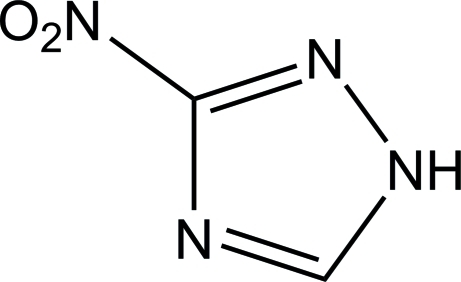


## Experimental

### 

#### Crystal data

C_2_H_2_N_4_O_2_
*M* *_r_* = 114.08
Monoclinic, 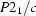

*a* = 8.7818 (1) Å
*b* = 10.0726 (2) Å
*c* = 9.9703 (1) Å
β = 107.081 (1)°
*V* = 843.03 (2) Å^3^
*Z* = 8
Mo *K*α radiation
μ = 0.16 mm^−1^
*T* = 100 K
0.48 × 0.33 × 0.30 mm

#### Data collection

Bruker SMART APEXII CCD area-detector diffractometer
Absorption correction: multi-scan (*SADABS*; Bruker, 2009 ▶) *T*_min_ = 0.928, *T*_max_ = 0.954
11450 measured reflections
3081 independent reflections
2768 reflections with *I* > 2σ(*I*)
*R*_int_ = 0.022

#### Refinement

*R*[*F* ^2^ > 2σ(*F* ^2^)] = 0.035
*wR*(*F* ^2^) = 0.092
*S* = 1.05
3081 reflections
153 parameters
H atoms treated by a mixture of independent and constrained refinement
Δρ_max_ = 0.50 e Å^−3^
Δρ_min_ = −0.40 e Å^−3^

### 

Data collection: *APEX2* (Bruker, 2009 ▶); cell refinement: *SAINT* (Bruker, 2009 ▶); data reduction: *SAINT*; program(s) used to solve structure: *SHELXTL* (Sheldrick, 2008 ▶); program(s) used to refine structure: *SHELXTL*; molecular graphics: *SHELXTL*; software used to prepare material for publication: *SHELXTL* and *PLATON* (Spek, 2009 ▶).

## Supplementary Material

Crystal structure: contains datablocks global, I. DOI: 10.1107/S1600536810049287/is2634sup1.cif

Structure factors: contains datablocks I. DOI: 10.1107/S1600536810049287/is2634Isup2.hkl

Additional supplementary materials:  crystallographic information; 3D view; checkCIF report

## Figures and Tables

**Table 1:** Hydrogen-bond geometry (Å, °)

*D*—H⋯*A*	*D*—H	H⋯*A*	*D*⋯*A*	*D*—H⋯*A*
N2*A*—H1*N*1⋯N1*A*^i^	0.885 (15)	1.995 (15)	2.8540 (9)	163.4 (15)
N2*B*—H1*N*2⋯N1*B*^ii^	0.857 (16)	2.057 (16)	2.9128 (10)	176.0 (16)
C1*A*—H1*AA*⋯O2*A*^iii^	0.93	2.50	3.1129 (10)	124
C1*B*—H1*BA*⋯O2*B*^ii^	0.93	2.51	3.0451 (11)	117

Symmetry codes: (i) 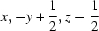; (ii) 
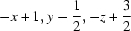
; (iii) 
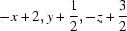
.

**Fig. 1.**
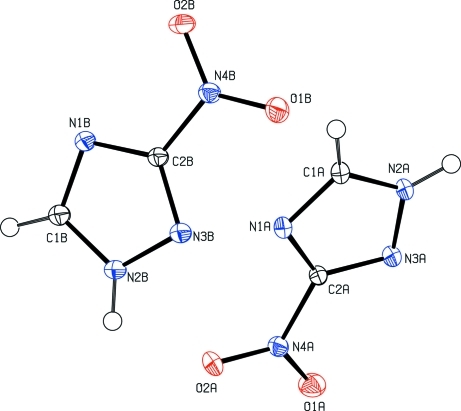
The asymmetric unit of the title compound. Displacement ellipsoids are drawn at the 50% probability level.

**Fig. 2.**
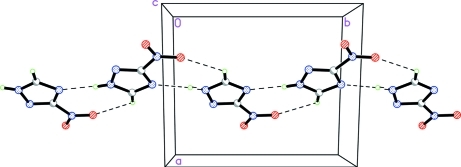
The crystal packing of the title compound, showing a hydrogen-bonded (dashed lines) molecular chain.

## supplementary crystallographic information

### Comment

1*H*-1,2,4-Triazole ring systems are typical planar six-π-electron partially aromatic systems, and are used, along with their derivatives, as starting materials for the synthesis of many heterocycles (Desenko, 1995). Substituted 1*H*-1,2,4-triazoles have also been actively studied as bridging ligands coordinating through their vicinal N atoms and some have special structures with interesting magnetic properties (Vos *et al.*, 1983; van Albada *et al.*, 1984).  Studies also indicate that the 1*H*-1,2,4-triazole system is associated with anticorrosion (Al-Kharafi *et al.*, 1986) and anti-inflammatory action (Gupta & Bhargava, 1978) and other pharmacological activities by exhibiting antiviral, anti-asthmatic, diuretic, analgesic, antimicrobial, antidepressant and antifungal effects (Jones *et al.*, 1965; Bennur *et al.*, 1976).

The asymmetric unit of the title compound consists of two crystallographically independent 3-nitro-1*H*-1,2,4-triazole molecules (A & B) with very similar geometry (Fig. 1).  The 1*H*-1,2,4-triazole units are essentially planar with maximum deviations of 0.003 (1) Å for atom N1A (molecule A) and 0.003 (1) Å for atom  C2B (molecule B). The dihedral angle between the two 1*H*-1,2,4-triazole (N1A—N3A/ C1A–C2A) and (N1B—N3B/C1B–C2B) rings is 56.58 (5)°.

In the crystal structure (Fig. 2), molecules are connected *via* N2A—H1N1···N1A, N2B—H1N2···N1B, C1A—H1AA···O2A and C1B—H1BA···O2B (Table 1) hydrogen bonds to form a one-dimensional supramolecular chain along the *b*-axis.

### Experimental

Hot methanol solution (20 ml) of 3-nitro-1*H*-1,2,4-triazole (57 mg, Aldrich) was warmed over a heating magnetic stirrer for 5 minutes. The resulting solution was allowed to cool slowly at room temperature. Crystals of the title compound appeared from the mother liquor after a few days.

### Refinement

Atoms H1N1 and H1N2 were located from a difference Fourier map and refined freely [refined N—H distances 0.857 (16) and 0.885 (15) Å]. The remaining H atoms were positioned geometrically [C—H = 0.93 Å] and were refined using a riding model, with *U*_iso_(H) = 1.2 *U*_eq_(C).

### Crystal data

**Table:**

C_2_H_2_N_4_O_2_	*F*(000) = 464
*M**_r_* = 114.08	*D*_x_ = 1.798 Mg m^−^^3^
Monoclinic, *P*2_1_/*c*	Mo *K*α radiation, λ = 0.71073 Å
Hall symbol: -P 2ybc	Cell parameters from 7180 reflections
*a* = 8.7818 (1) Å	θ = 2.9–32.6°
*b* = 10.0726 (2) Å	µ = 0.16 mm^−^^1^
*c* = 9.9703 (1) Å	*T* = 100 K
β = 107.081 (1)°	Block, colourless
*V* = 843.03 (2) Å^3^	0.48 × 0.33 × 0.30 mm
*Z* = 8	

### Data collection

**Table:**

Bruker SMART APEXII CCD area-detector diffractometer	3081 independent reflections
Radiation source: fine-focus sealed tube	2768 reflections with *I* > 2σ(*I*)
graphite	*R*_int_ = 0.022
φ and ω scans	θ_max_ = 32.7°, θ_min_ = 2.9°
Absorption correction: multi-scan (*SADABS*; Bruker, 2009)	*h* = −13→11
*T*_min_ = 0.928, *T*_max_ = 0.954	*k* = −15→13
11450 measured reflections	*l* = −15→15

### Refinement

**Table:**

Refinement on *F*^2^	Primary atom site location: structure-invariant direct methods
Least-squares matrix: full	Secondary atom site location: difference Fourier map
*R*[*F*^2^ > 2σ(*F*^2^)] = 0.035	Hydrogen site location: inferred from neighbouring sites
*wR*(*F*^2^) = 0.092	H atoms treated by a mixture of independent and constrained refinement
*S* = 1.05	*w* = 1/[σ^2^(*F*_o_^2^) + (0.0495*P*)^2^ + 0.2412*P*] where *P* = (*F*_o_^2^ + 2*F*_c_^2^)/3
3081 reflections	(Δ/σ)_max_ = 0.001
153 parameters	Δρ_max_ = 0.50 e Å^−^^3^
0 restraints	Δρ_min_ = −0.40 e Å^−^^3^

### Special details

**Table:**

Experimental. The crystal was placed in the cold stream of an Oxford Cryosystems Cobra open-flow nitrogen cryostat (Cosier & Glazer, 1986) operating at 100.0 (1) K.
Geometry. All s.u.'s (except the s.u. in the dihedral angle between two l.s. planes) are estimated using the full covariance matrix. The cell s.u.'s are taken into account individually in the estimation of s.u.'s in distances, angles and torsion angles; correlations between s.u.'s in cell parameters are only used when they are defined by crystal symmetry. An approximate (isotropic) treatment of cell s.u.'s is used for estimating s.u.'s involving l.s. planes.
Refinement. Refinement of F^2^ against ALL reflections. The weighted R-factor wR and goodness of fit S are based on F^2^, conventional R-factors R are based on F, with F set to zero for negative F^2^. The threshold expression of F^2^ > 2σ(F^2^) is used only for calculating R-factors(gt) etc. and is not relevant to the choice of reflections for refinement. R-factors based on F^2^ are statistically about twice as large as those based on F, and R- factors based on ALL data will be even larger.

### Fractional atomic coordinates and isotropic or equivalent isotropic displacement parameters (Å^2^)

**Table:**

	*x*	*y*	*z*	*U*_iso_*/*U*_eq_	
O1A	0.73082 (8)	−0.04055 (7)	0.51717 (7)	0.01914 (13)	
O2A	0.85716 (9)	0.01680 (7)	0.73151 (6)	0.01888 (14)	
N1A	1.01266 (8)	0.21389 (7)	0.64087 (7)	0.01307 (13)	
N2A	0.99857 (9)	0.24376 (7)	0.42001 (7)	0.01273 (13)	
N3A	0.89747 (8)	0.14183 (7)	0.41773 (7)	0.01286 (13)	
N4A	0.82618 (9)	0.02813 (7)	0.60358 (7)	0.01348 (13)	
C1A	1.06543 (10)	0.28585 (8)	0.55150 (8)	0.01356 (14)	
H1AA	1.1381	0.3552	0.5768	0.016*	
C2A	0.91199 (9)	0.13008 (8)	0.55271 (8)	0.01167 (14)	
O1B	0.75840 (8)	0.41600 (7)	0.50676 (7)	0.02046 (14)	
O2B	0.68377 (9)	0.58439 (6)	0.60867 (7)	0.01985 (14)	
N1B	0.51771 (8)	0.42579 (7)	0.73353 (7)	0.01312 (13)	
N2B	0.51813 (9)	0.20833 (7)	0.72132 (7)	0.01419 (13)	
N3B	0.60998 (9)	0.24714 (7)	0.64058 (7)	0.01375 (13)	
N4B	0.68913 (8)	0.46461 (7)	0.58504 (7)	0.01361 (13)	
C1B	0.46484 (10)	0.31423 (8)	0.77581 (8)	0.01436 (15)	
H1BA	0.4002	0.3102	0.8347	0.017*	
C2B	0.60451 (9)	0.37710 (8)	0.65365 (8)	0.01189 (14)	
H1N1	1.0120 (19)	0.2722 (16)	0.3403 (16)	0.034 (4)*	
H1N2	0.5028 (18)	0.1259 (16)	0.7343 (15)	0.030 (4)*	

### Atomic displacement parameters (Å^2^)

**Table:**

	*U*^11^	*U*^22^	*U*^33^	*U*^12^	*U*^13^	*U*^23^
O1A	0.0195 (3)	0.0170 (3)	0.0203 (3)	−0.0044 (2)	0.0049 (2)	−0.0008 (2)
O2A	0.0278 (3)	0.0179 (3)	0.0136 (3)	0.0007 (2)	0.0102 (2)	0.0037 (2)
N1A	0.0164 (3)	0.0135 (3)	0.0103 (3)	−0.0005 (2)	0.0055 (2)	−0.0003 (2)
N2A	0.0164 (3)	0.0133 (3)	0.0099 (3)	0.0001 (2)	0.0060 (2)	0.0011 (2)
N3A	0.0156 (3)	0.0128 (3)	0.0107 (3)	0.0004 (2)	0.0048 (2)	0.0009 (2)
N4A	0.0161 (3)	0.0118 (3)	0.0144 (3)	0.0020 (2)	0.0072 (2)	0.0018 (2)
C1A	0.0163 (3)	0.0142 (3)	0.0115 (3)	−0.0007 (3)	0.0060 (3)	−0.0005 (2)
C2A	0.0146 (3)	0.0109 (3)	0.0107 (3)	0.0013 (2)	0.0056 (2)	0.0010 (2)
O1B	0.0240 (3)	0.0185 (3)	0.0245 (3)	0.0046 (2)	0.0158 (3)	0.0025 (2)
O2B	0.0264 (3)	0.0106 (3)	0.0260 (3)	−0.0006 (2)	0.0131 (3)	0.0004 (2)
N1B	0.0151 (3)	0.0111 (3)	0.0143 (3)	0.0010 (2)	0.0062 (2)	0.0004 (2)
N2B	0.0178 (3)	0.0101 (3)	0.0161 (3)	−0.0002 (2)	0.0072 (2)	0.0006 (2)
N3B	0.0163 (3)	0.0111 (3)	0.0149 (3)	0.0009 (2)	0.0062 (2)	0.0007 (2)
N4B	0.0144 (3)	0.0119 (3)	0.0153 (3)	0.0015 (2)	0.0055 (2)	0.0018 (2)
C1B	0.0166 (3)	0.0119 (3)	0.0160 (3)	0.0006 (3)	0.0070 (3)	0.0005 (2)
C2B	0.0128 (3)	0.0103 (3)	0.0127 (3)	0.0007 (2)	0.0041 (2)	0.0007 (2)

### Geometric parameters (Å, °)

**Table:**

O1A—N4A	1.2241 (10)	O1B—N4B	1.2239 (9)
O2A—N4A	1.2289 (9)	O2B—N4B	1.2329 (9)
N1A—C1A	1.3329 (10)	N1B—C1B	1.3307 (10)
N1A—C2A	1.3455 (10)	N1B—C2B	1.3462 (10)
N2A—C1A	1.3383 (10)	N2B—C1B	1.3421 (10)
N2A—N3A	1.3531 (10)	N2B—N3B	1.3539 (9)
N2A—H1N1	0.885 (15)	N2B—H1N2	0.857 (16)
N3A—C2A	1.3194 (9)	N3B—C2B	1.3178 (10)
N4A—C2A	1.4506 (10)	N4B—C2B	1.4476 (10)
C1A—H1AA	0.9300	C1B—H1BA	0.9300
			
C1A—N1A—C2A	101.26 (6)	C1B—N1B—C2B	100.99 (7)
C1A—N2A—N3A	110.72 (6)	C1B—N2B—N3B	110.52 (7)
C1A—N2A—H1N1	129.9 (10)	C1B—N2B—H1N2	128.3 (10)
N3A—N2A—H1N1	119.4 (10)	N3B—N2B—H1N2	121.2 (10)
C2A—N3A—N2A	100.64 (6)	C2B—N3B—N2B	100.52 (6)
O1A—N4A—O2A	125.11 (7)	O1B—N4B—O2B	124.56 (7)
O1A—N4A—C2A	118.18 (6)	O1B—N4B—C2B	118.56 (7)
O2A—N4A—C2A	116.70 (7)	O2B—N4B—C2B	116.86 (6)
N1A—C1A—N2A	110.12 (7)	N1B—C1B—N2B	110.33 (7)
N1A—C1A—H1AA	124.9	N1B—C1B—H1BA	124.8
N2A—C1A—H1AA	124.9	N2B—C1B—H1BA	124.8
N3A—C2A—N1A	117.27 (7)	N3B—C2B—N1B	117.63 (7)
N3A—C2A—N4A	121.04 (7)	N3B—C2B—N4B	121.29 (7)
N1A—C2A—N4A	121.66 (6)	N1B—C2B—N4B	121.08 (7)
			
C1A—N2A—N3A—C2A	−0.05 (8)	C1B—N2B—N3B—C2B	0.10 (9)
C2A—N1A—C1A—N2A	−0.45 (9)	C2B—N1B—C1B—N2B	−0.54 (9)
N3A—N2A—C1A—N1A	0.33 (10)	N3B—N2B—C1B—N1B	0.30 (10)
N2A—N3A—C2A—N1A	−0.27 (9)	N2B—N3B—C2B—N1B	−0.49 (9)
N2A—N3A—C2A—N4A	−178.46 (7)	N2B—N3B—C2B—N4B	179.31 (7)
C1A—N1A—C2A—N3A	0.46 (9)	C1B—N1B—C2B—N3B	0.66 (9)
C1A—N1A—C2A—N4A	178.64 (7)	C1B—N1B—C2B—N4B	−179.14 (7)
O1A—N4A—C2A—N3A	−5.31 (11)	O1B—N4B—C2B—N3B	4.58 (12)
O2A—N4A—C2A—N3A	173.84 (7)	O2B—N4B—C2B—N3B	−176.50 (8)
O1A—N4A—C2A—N1A	176.57 (7)	O1B—N4B—C2B—N1B	−175.62 (8)
O2A—N4A—C2A—N1A	−4.27 (11)	O2B—N4B—C2B—N1B	3.29 (11)

### Hydrogen-bond geometry (Å, °)

**Table:**

*D*—H···*A*	*D*—H	H···*A*	*D*···*A*	*D*—H···*A*
N2A—H1N1···N1A^i^	0.885 (15)	1.995 (15)	2.8540 (9)	163.4 (15)
N2B—H1N2···N1B^ii^	0.857 (16)	2.057 (16)	2.9128 (10)	176.0 (16)
C1A—H1AA···O2A^iii^	0.93	2.50	3.1129 (10)	124.
C1B—H1BA···O2B^ii^	0.93	2.51	3.0451 (11)	117.

Symmetry codes: (i) *x*, −*y*+1/2, *z*−1/2; (ii) −*x*+1, *y*−1/2, −*z*+3/2; (iii) −*x*+2, *y*+1/2, −*z*+3/2.
